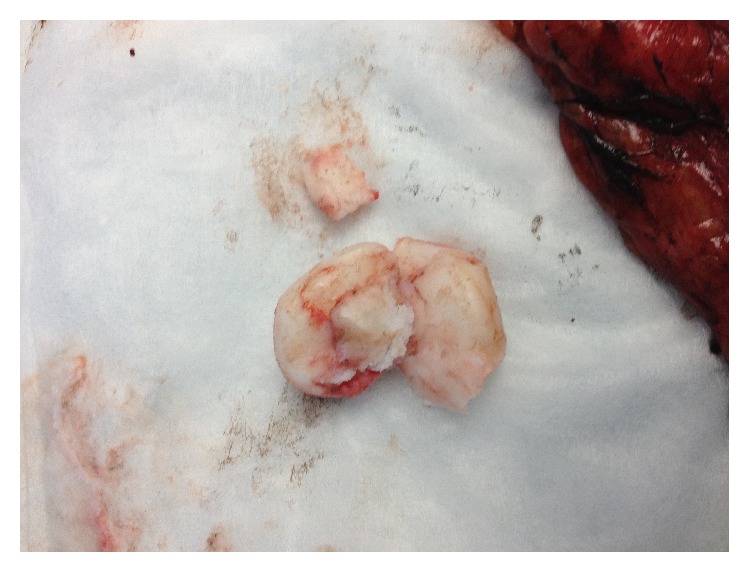# A Case Report and Review of the Literature of Adult Gastric Duplication Cyst

**DOI:** 10.1155/2015/240891

**Published:** 2015-05-21

**Authors:** Scott Samona, Richard Berri

**Affiliations:** General Surgery Department, St. John Hospital and Medical Center, Detroit, MI 48236, USA

## Abstract

Gastrointestinal (GI) duplication cysts are a rare congenital disease. They may involve any level of the alimentary tract, but they most commonly involve the ileum, esophagus, and jejunum. Gastric duplication cysts represent approximately 4–8% of GI duplication cysts, the majority of which present in early childhood. We present a rare case of adult gastric duplication cyst in a 25-year-old female found to have abdominal mass on computed tomography imaging. There are several potential methods to diagnose gastric duplication cyst and treatment of choice is complete surgical resection.

## 1. Introduction

Gastrointestinal duplication cysts are a rare congenital disease. They tend to be hollow, spherical, or tubular structures, with well-developed smooth muscle coats, lined by mucosal epithelium. These structures tend to develop prior to complete differentiation of gastrointestinal epithelium and as such are often named after their organ of association [1, 2]. The most common gastrointestinal duplication cysts are those that involve the ileum, esophagus, and jejunum [3, 4]. Being highly uncommon, gastric duplication cysts represent approximately 4–8% of all gastrointestinal duplication cysts [5]. The majority of gastric duplication cysts present in early childhood with 67% identified within the first year of life [6]. We report an adult case of gastric duplication cyst in a 25-year-old female.

## 2. Case Presentation

**Figure 1:**
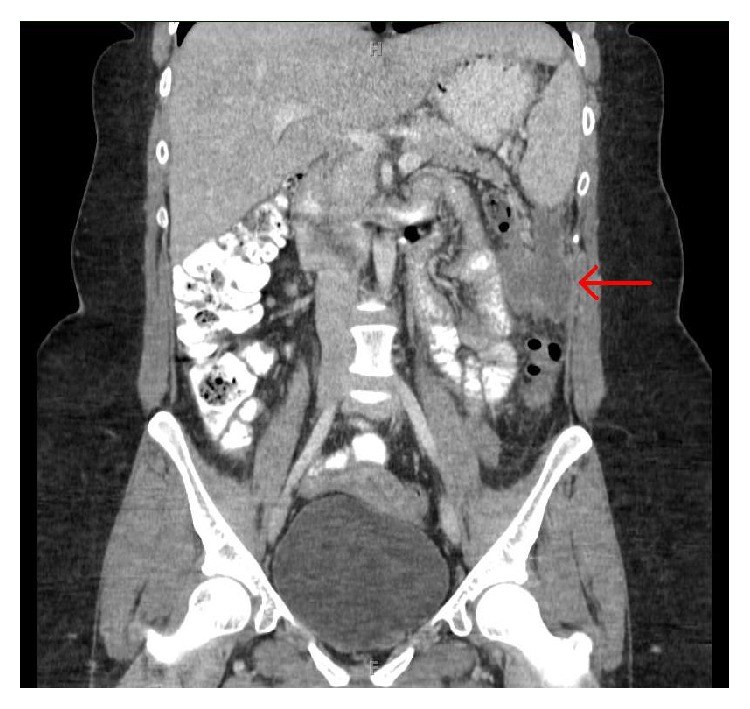


**Figure 2:**
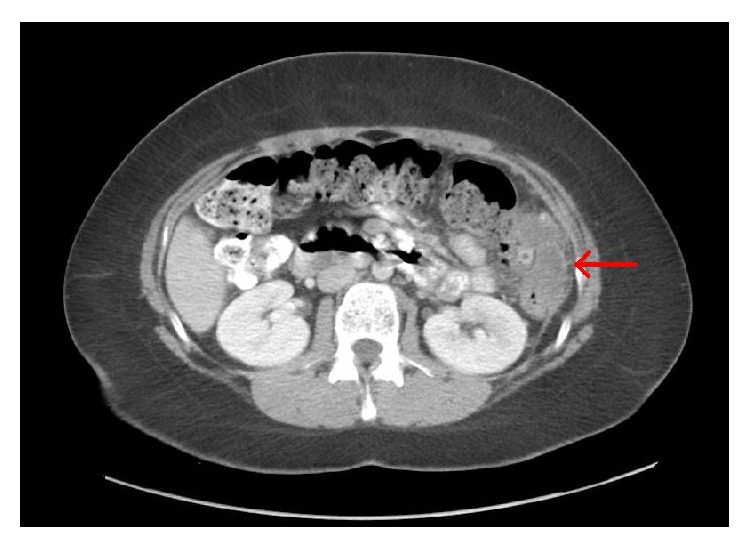


The patient is a 25-year-old female, with no significant previous medical or surgical history, who presented to an outside facility after experiencing approximately one week of abdominal pain of acute onset. Pain was described as primarily mid-abdominal in origin with sharp quality but gradually became diffuse and colicky in nature. The patient also experienced associated nausea and bilious vomiting. There were no changes in bowel habits. Patient denied coffee ground emesis, hematemesis, melena, or hematochezia. She also denied any recent history of weight loss or weight gain. Differential diagnoses at this time included gallbladder disease, reflux esophagitis, gastritis, peptic ulcer disease, acute pancreatitis, and partial bowel obstruction. The patient underwent diagnostic evaluation by computed tomography (CT), which demonstrated a cystic-appearing lesion near the splenic flexure measuring 7.6 cm cranial-caudal × 4.1 cm transverse × 4.3 cm anterior-posterior (Figures 1 and 2). CT-guided fine needle aspiration was performed, of which pathology demonstrated mucinous fluid with mucus epithelium, concerning potential mucinous neoplasm of either gastrointestinal or gynecologic origin. The patient's CT scan demonstrated normal architecture of the colon, appendix, uterus, and ovaries; however additional diagnostic evaluation was performed. The patient underwent transvaginal ultrasonography of the uterus and ovaries, which was unremarkable. She also underwent total colonoscopy without any evidence of malignancy. CA 19-9, CA-125, and CEA levels were all found to be within normal limits.

The patient subsequently underwent diagnostic laparoscopy demonstrating evidence of dense adhesions within the left upper quadrant; however they appeared inflammatory versus malignant in nature. There was no evidence of carcinomatosis, ascites, or liver metastases. There was evidence of a localized mass versus inflammatory reaction within the omentum in the left upper quadrant near the spleen and tail of the pancreas. Conversion to open exploratory laparotomy was performed and the patient underwent debulking of tumor with omentectomy and partial pancreatectomy. The gross specimen (Figure 3) with omental adhesions was closely examined. The specimen was evaluated under frozen section by our institution's pathology team; however no evidence of malignancy was identified by histologic examination. At this time the operation was completed and the specimen was sent to an outside facility for further analysis by expert gastrointestinal pathologist. Final pathology of the specimen demonstrated a 3.1 × 2.7 × 2.6 cm cyst with mucinous epithelial lining (foveolar type) and prominent smooth muscle with evidence of inflammation. Further examination established diagnosis of gastric duplication cyst.

The patient was seen at one-month follow-up and had an uncomplicated postoperative course. She was found to be doing well with no complaints. Abdominal pain had completely resolved and she was able to tolerate a regular diet without nausea or vomiting. She continued to do well and was instructed to follow up in one year's time.

## 3. Discussion

Duplication cysts of the gastrointestinal tract are relatively rare phenomena with the majority occurring in the ileum [3, 4] and rarely involving the stomach [5]. Established criteria for diagnosis of gastric duplication cyst include the wall of the cyst being contiguous with the stomach wall, the presence of smooth muscle surrounding the cyst and in continuation with the gastric musculature, and lining of the cyst wall by epithelial, gastric, or gut mucosa of any type [1, 2, 6, 7]. These lesions are thought to be congenital in nature and develop prior to complete differentiation of the gastrointestinal epithelium. As such they are named after the organ of association [1, 2, 8].

Because of the potential for neoplastic transformation, it is recommended that duplication cysts be surgically excised when found [9]. These malformations have been associated with development of carcinoma and adenomyoma [6]. As most of these lesions remain asymptomatic until becoming large enough to cause compressive symptoms, most present after significant associated inflammatory reaction has taken place. Although preoperative diagnosis may be difficult, various diagnostic techniques may be utilized. CT imaging as well as endoscopic ultrasound may be of use for establishing location, size, and local tissue involvement. Specific radiographic features may aid in diagnosis. Gastric duplication cysts typically appear as cystic lesions with thick walls and often exhibit contrast enhancement of the inner lining on CT examination. These lesions may also demonstrate calcifications; however this may mimic pancreatic cystic tumors if in close proximity to the pancreas [10]. Endoscopic ultrasound may also provide some aid in diagnosis. Features of gastric duplication cysts include cystic lesion with hypoechoic muscle layer and echogenic internal mucosal layer [2, 10]. Additional radiographic techniques, such as magnetic resonance imaging, may also be of use in select patients.

In conclusion, although rare, gastrointestinal duplication cysts are unique entities that are often detected after significant growth with development of compressive symptoms. They are often diagnosed postoperatively by careful pathologic examination, and treatment of choice is complete surgical resection.

## Figures and Tables

**Figure 3:**